# Interaction of lactate/albumin and geriatric nutritional risk index on the all‐cause mortality of elderly patients with critically ill heart failure: A cohort study

**DOI:** 10.1002/clc.24029

**Published:** 2023-05-24

**Authors:** Wanli Chen, Meixia Chen, Xiao Qiao

**Affiliations:** ^1^ Department of General Practice Bengbu Third People's Hospital Bengbu People's Republic of China

**Keywords:** critically ill elderly patients, GNRI, heart failure, interaction effect, L/A ratio, nutritional status

## Abstract

**Background:**

Whether there is a multiplicative interaction of lactate/albumin (L/A) ratio and geriatric nutritional risk index (GNRI) on the mortality of critically ill elderly patients with heart failure (HF) remains unclear.

**Hypothesis:**

To assess the interaction of L/A ratio and GNRI on the all‐cause mortality in critically ill elderly patients with HF.

**Methods:**

This was a retrospective cohort study and data were extracted from the Medical Information Mart for Intensive Care III (MIMIC‐III) database. The endpoints were 28‐day and 1‐year all‐cause mortality, and the independent variables were L/A ratio and GNRI. The multiplicative interaction of L/A ratio and GNRI on the mortality was examined using Cox proportional‐hazards model.

**Results:**

A total of 5627 patients were finally included. Results showed that patients with higher L/A ratio or GNRI ≤ 58 had higher risk of 28‐day and 1‐year all‐cause mortality (all *p* < .01). We also found the significant multiplicative interaction effect between L/A ratio and GNRI score on the 28‐day and 1‐year all‐cause mortality (both *p* < .05). The increased L/A ratio was associated with higher risk of 28‐day and 1‐year all‐cause mortality in patients with GNRI ≤ 58 than those with GNRI > 58.

**Conclusions:**

There was a multiplicative interaction effect between L/A ratio and GNRI score on the mortality, and low GNRI score was associated with the increased risk of all‐cause mortality with the increase of L/A ratio, suggesting the importance of nutrition‐oriented intervention in critically ill elderly HF patients with high L/A ratio.

## Graphical abstract

Graphical abstract showed that high lactate/albumin (L/A) ratio or geriatric nutritional risk index (GNRI) ≤ 58 was associated with the high risk of 28‐day and 1‐year all‐cause mortality. There was a significant multiplicative interaction effect between L/A ratio and GNRI score on the 28‐day and 1‐year all‐cause mortality.

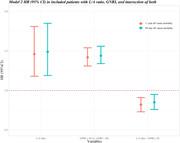

## 1 INTRODUCTION

Heart failure (HF), a syndrome caused by heart function disorder, is a main reason of cardiovascular morbidity and mortality.^1^ The prevalence of HF ranges from approximately 1% in middle‐aged people to more than 10% in elderly people.^2^ About 25.5% of critically ill patients with HF died within 1 year after hospital discharge.^3^ Evidence has showed that the mortality of HF is age‐dependent, and 1‐year mortality in the elderly reaches up to 7.4% for 60‐year‐olds and 19.5% for 80‐year‐olds, which poses an overwhelming threat to human health and leads to a huge medical burden.^4^ To decrease the mortality of critically ill elderly patients with HF, it is important to identify those at the high risk of mortality.

The elevated blood lactate is considered as a marker of tissue hypoperfusion in critical care medicine and used as a prognostic marker for helping make therapeutic decision.^5^ However, lactate elevation may be caused by various conditions, including reduced lactate elimination due to renal or hepatic dysfunction and accelerated glycolysis; thus, predictive capacity solely based on the original lactate level might be low.^6^ In addition to lactate, serum albumin, which acts as a negative acute‐phase protein and reflects the severity of inflammation, has been reported as a factor to predict the mortality in HF patients.^7^ However, albumin levels may be impacted by chronic inflammatory conditions and nutritional status.^8^ Therefore, only using albumin level to predict the prognosis may have limitations. The lactate/albumin (L/A) ratio is believed to be a more effective prognostic marker than lactate or albumin alone for intensive care patients.^9^ In critically ill adults with HF, L/A ratio has been confirmed as an important predictor of short‐term and long‐term mortality, while the diagnostic utility of L/A ratio in elderly patients has not been reported.^10^

The nutritional status in critically ill patients is a common concern, and it is worth noting that the incidence of malnutrition among elderly patients is 71.24%, which is far higher than 28% among younger patients.^11^ Previous studies have reported that malnutrition is a useful predictor of mortality in HF patients.^12^, ^13^, ^14^ Geriatric nutritional risk index (GNRI), comprising three objective parameters (height, weight, and serum albumin), is a simple tool to assess the nutritional status in the elderly.^12^ A clinical study has reported the superiority of GNRI to other scores in predicting short‐term mortality for elderly HF patients.^15^ Nishi et al. reports that nutritional screening using the GNRI is contributive to predict the long‐term mortality of elderly HF patients.^16^ Currently, although L/A ratio and GNRI has been confirmed as independent predictors of mortality in HF, whether there is a multiplicative interaction effect of L/A ratio and GNRI on the mortality of critically ill elderly patients with HF remains unclear.

Therefore, we designed a cohort study to evaluate the association and multiplicative interaction of L/A ratio and GNRI on the short‐term and long‐term mortality in critically ill elderly patients with HF.

## 2 METHODS

### 2.1 Study design and data source

This is a retrospective cohort study based on the Medical Information Mart for Intensive Care III (MIMIC‐III) database (version 1.4). MIMIC‐III is a large and single‐center database including health‐related data on over 40 000 patients who are admitted to the intensive care unit (ICU) of Beth Israel Deaconess Medical Center from 2001 to 2012.^17^ This database includes comprehensive clinical data, containing demographic characteristics, vital signs, laboratory measurements, international classification of diseases‐ninth revision (ICD‐9) codes, medication, and medical records. MIMIC‐III is a freely accessible database and all data are deidentified; thus, this study is exempt from written informed consent of patients. The project has obtained approval from Beth Israel Deaconess Medical Center and the Institutional Review Board of the Massachusetts Institute of Technology. This study was performed according to the revised Declaration of Helsinki in 2013.

### 2.2 Study population

We included critically ill elderly patients with HF (age above 70 years) at first ICU admission and stayed more than 48 h in the hospital. Patients who missed basic data on height, weight, lactate, and albumin, and had extreme height (height = 0 cm, height < 100 cm while weight > 55 kg, or height > 300 cm) were excluded from this study.

### 2.3 Endpoints

Our study endpoints were 28‐day and 1‐year all‐cause mortality after the date of ICU admission. From the Social Security Death Index records, survival information containing survival outcome and time of death was extracted. Patients were followed up until they were dead.

### 2.4 Independent variables

The independent variables in our study were L/A ratio and GNRI. According to the quintile (Q) of L/A ratio, we stratified the participants into five groups: Q1 (L/A ratio ≤ 0.31), Q2 (0.31 < L/A ratio ≤ 0.43), Q3 (0.43 < L/A ratio ≤ 0.58), Q4 (0.58 < L/A ratio ≤ 0.87), and Q5 (L/A ratio > 0.87).

GNRI formula was shown as: GNRI = [1.489 × albumin (g/L)] + [41.7 × (body weight (kg)/ideal body weight (kg)].^18^ The ideal body weight (kg) deriving from Lorentz equations for men was height (cm) − 100 − (height − 150/4) and that for women was height (cm) − 100 − (height − 150/2.5). Body weight/ideal body weight ratio was expressed as 1 if the patient's body weight was greater than the ideal weight. We used the restricted cubic spline (RCS) method to determine the cutoff value of GNRI in this study. According to the knots of curve, patients were divided into two groups: GNRI ≤ 58 and GNRI > 58 (Supporting Information: Figure 1A,B).

### 2.5 Covariates of interest

The covariates of interest included the following six aspects: (1) demographic characteristics: gender, age, marital status, ethnicity, body mass index (BMI); (2) medications: β‐blocker, angiotensin‐converting‐enzyme inhibitor (ACEI), angiotensin receptor blocker (ARB), diuretics; (3) comorbidities: peripheral vascular disease (PVD), hypertension, chronic obstructive pulmonary disease (COPD), diabetes, renal failure, atrial fibrillation (AF), myocardial infarction (MI); (4) vital signs: respiratory rate, heart rate, systolic blood pressure (SBP), diastolic blood pressure (DBP), and saturation of peripheral oxygen (SPO_2_); (5) laboratory measurements: hemoglobin, neutrophils, lymphocytes, hematocrit, albumin, blood urea nitrogen (BUN), creatinine, creatine kinase, potassium, sodium, pH, lactate, glucose, partial pressure of oxygen (PO_2_), partial pressure of carbon dioxide (PCO_2_), base excess (BE); (6) severity of disease: Glasgow Coma Scale (GCS), Oxford Acute Severity of Illness (OASIS), Simplified Acute Physiology Score (SAPS), and Sequential Organ Failure Assessment (SOFA). The variables with statistical differences among groups stratified by L/A ratio or by GNRI score were selected, and stepwise regression method was adopted to further screen the variables. The finally included variables into multivariate Cox proportional‐hazards model were age, marital status, ethnicity, BMI, β‐blocker, ACEI, ARB, hypertension, diabetes, renal failure, hemoglobin, hematocrit, lymphocytes, BUN, creatinine, creatine kinase, sodium, glucose, PO_2_, BE, GCS, OASIS, SAPS, SOFA.

### 2.6 Statistical analysis

The continuous variables were presented as mean ± standard deviation (mean ± SD), and categorical variables were presented as number (*n*) and percentage (%). For groups divided by L/A ratio, differences of continuous variables were compared using analysis of variance or Kruskal–Wallis test and differences of categorical variables were compared using *χ* ^2^ test or Fisher's exact test. For groups stratified by GNRI score, differences of continuous variables were compared using Student's *t* test (normally distributed), and categorical variables were compared using *χ* ^2^ test or Fisher's exact test. Missing data were imputed using multiple imputations. The cumulative 28‐day and 1‐year all‐cause mortality by L/A ratio quintiles and GNRI score group was shown through Kaplan–Meier (KM) method. Cut‐off point of GNRI score was determined using the RCS method. Univariate and multivariate Cox proportional‐hazards models were used to examine the association and multiplicative interaction of L/A ratio and GNRI score on the 28‐day and 1‐year all‐cause mortality, and results were expressed as hazard ratio (HR) with 95% confidence interval (95% CI). A two‐sided *p* < .05 was considered to be statistically significant. All statistical analyses were performed using R (v4.1.2, R Foundation for Statistical Computing).

## 3 RESULTS

### 3.1 Selection and characteristics of patients

**Figure 1:**
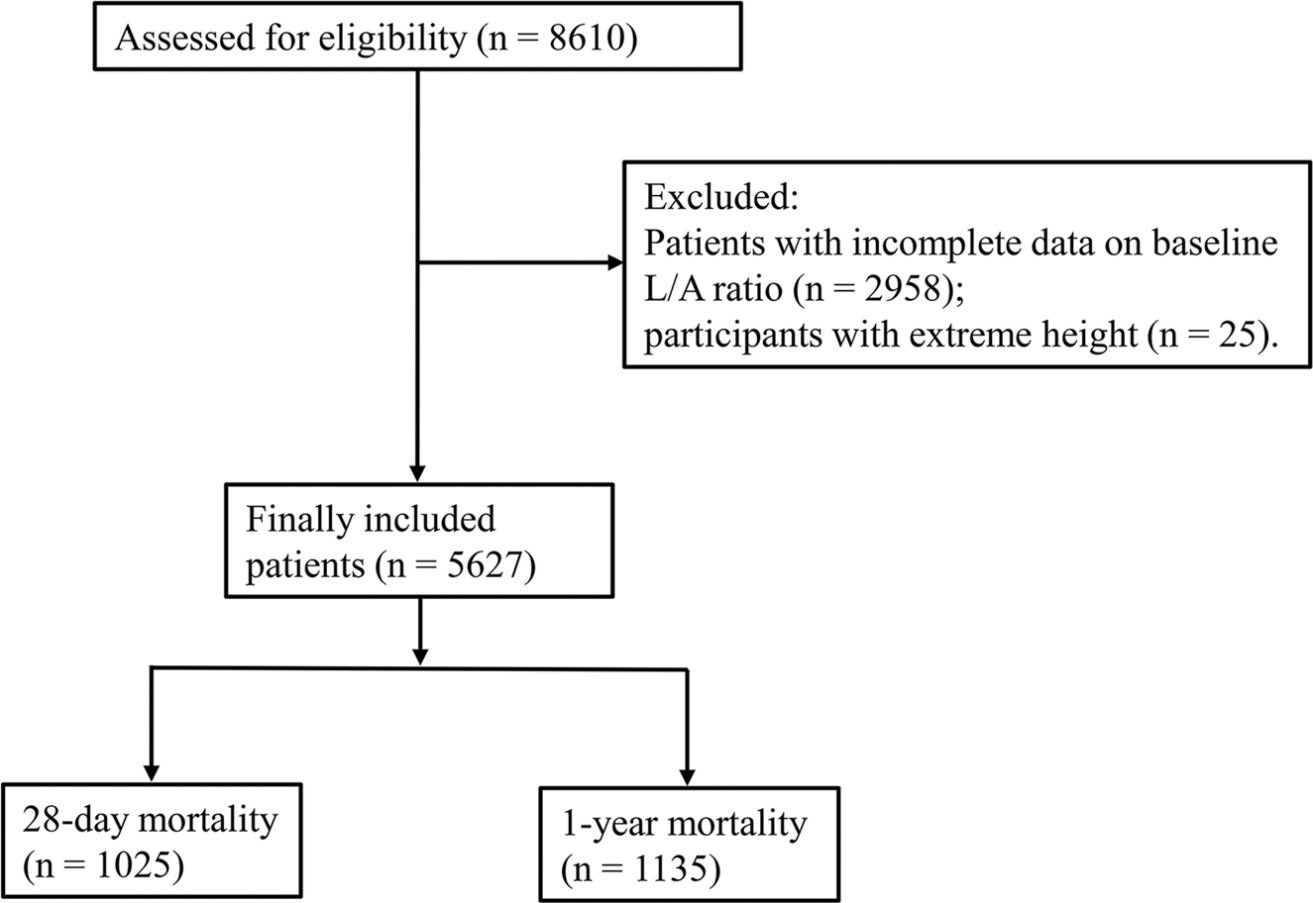
The flowchart of selecting patients.

Overall, 8610 critically ill elderly patients with HF were assessed for eligibility according to the inclusion criteria. Of these, 2958 patients missing data on baseline L/A ratio and 25 patients with extreme height were excluded. Finally, 5627 patients were included for analysis. There were 1025 patients in the 28‐day all‐cause mortality group and 1135 patients in the all‐cause 1‐year mortality group (Figure 1).

The mean age of overall patients was 70.85 (13.27) years, and there were 59.2% of male (*n* = 3332). Characteristics of patients stratified by L/A ratio and GNRI score were shown in Table 1. There was a significant difference in marital status, ethnicity, BMI, β‐blocker, ACEI, ARB, diuretics, hypertension, COPD, AF, respiratory rate, heart rate, SBP, DBP, hemoglobin, neutrophil, lymphocytes, albumin, BUN, creatinine, creatine kinase, potassium, sodium, pH, lactate, glucose, PO_2_, PCO_2_, BE, GCS, OASIS, SAPS, and SOFA among the groups divided by L/A ratio. Gender, age, marital status, ethnicity, BMI, ARB, diuretics, PVD, COPD, diabetes, renal failure, hemoglobin, neutrophils, hematocrit, albumin, pH, lactate, glucose, PCO_2_, BE, OASIS, and SAPS were statistically different between groups divided by GNRI score.

**Table 1:** The characteristic of included patients.

Variable	Overall (*n* = 5627)	L/A ratio						GNRI score		
		Q1 (*n* = 1130)	Q2 (*n* = 1138)	Q3 (*n* = 1109)	Q4 (n = 1127)	Q5 (*n* = 1123)	*p*	GNRI > 58 (*n* = 2658)	GNRI ≤ 58 (*n* = 2969)	*p*
Gender, *n* (%)							.757			.042
Female	2295 (40.8)	467 (41.3)	462 (40.6)	464 (41.8)	462 (41.0)	440 (39.2)		1122 (42.2)	1173 (39.5)	
Male	3332 (59.2)	663 (58.7)	676 (59.4)	645 (58.2)	665 (59.0)	683 (60.8)		1536 (57.8)	1796 (60.5)	
Age (year), mean (SD)	70.85 (13.27)	70.95 (12.69)	70.76 (13.02)	70.28 (13.41)	71.61 (13.32)	70.64 (13.85)	.189	68.64 (12.57)	72.83 (13.55)	<.001
Marital status, *n* (%)							.032			<.001
Unknown	474 (8.4)	83 (7.3)	77 (6.8)	95 (8.6)	107 (9.5)	112 (10.0)		227 (8.5)	247 (8.3)	
Divorced	471 (8.4)	93 (8.2)	97 (8.5)	84 (7.6)	101 (9.0)	96 (8.5)		237 (8.9)	234 (7.9)	
Married	2532 (45.0)	534 (47.3)	551 (48.4)	510 (46.0)	472 (41.9)	465 (41.4)		1177 (44.3)	1355 (45.6)	
Single	1225 (21.8)	230 (20.4)	234 (20.6)	232 (20.9)	259 (23.0)	270 (24.0)		637 (24.0)	588 (19.8)	
Widowed	925 (16.4)	190 (16.8)	179 (15.7)	188 (17.0)	188 (16.7)	180 (16.0)		380 (14.3)	545 (18.4)	
Ethnicity, *n* (%)							<.001			<.001
White	18 (0.3)	2 (0.2)	3 (0.3)	5 (0.5)	6 (0.5)	2 (0.2)		13 (0.5)	5 (0.2)	
American Indian/Alaska	130 (2.3)	20 (1.8)	26 (2.3)	28 (2.5)	22 (2.0)	34 (3.0)		27 (1.0)	103 (3.5)	
Asian	531 (9.4)	81 (7.2)	90 (7.9)	107 (9.6)	111 (9.8)	142 (12.6)		238 (9.0)	293 (9.9)	
Black/African American	185 (3.3)	39 (3.5)	29 (2.5)	42 (3.8)	25 (2.2)	50 (4.5)		91 (3.4)	94 (3.2)	
Hispanic/Latino	245 (4.4)	59 (5.2)	47 (4.1)	44 (4.0)	43 (3.8)	52 (4.6)		112 (4.2)	133 (4.5)	
Others	83 (1.5)	20 (1.8)	26 (2.3)	6 (0.5)	12 (1.1)	19 (1.7)		37 (1.4)	46 (1.5)	
Unable to obtain	673 (12.0)	117 (10.4)	108 (9.5)	140 (12.6)	155 (13.8)	153 (13.6)		315 (11.9)	358 (12.1)	
Unknown	3762 (66.9)	792 (70.1)	809 (71.1)	737 (66.5)	753 (66.8)	671 (59.8)		1825 (68.7)	1937 (65.2)	
BMI (kg/m^2^), mean (SD)	29.18 (7.79)	29.11 (7.41)	29.48 (8.07)	29.50 (8.03)	29.29 (8.04)	28.50 (7.33)	0.014	35.07 (7.25)	23.90 (2.96)	<.001
β‐blocker, *n* (%)							<.001			.617
No	941 (16.7)	134 (11.9)	133 (11.7)	157 (14.2)	211 (18.7)	306 (27.2)		437 (16.4)	504 (17.0)	
Yes	4686 (83.3)	996 (88.1)	1005 (88.3)	952 (85.8)	916 (81.3)	817 (72.8)		2221 (83.6)	2465 (83.0)	
ACEI, *n* (%)							<.001			.679
No	3696 (65.7)	689 (61.0)	687 (60.4)	695 (62.7)	775 (68.8)	850 (75.7)		1738 (65.4)	1958 (65.9)	
Yes	1931 (34.3)	441 (39.0)	451 (39.6)	414 (37.3)	352 (31.2)	273 (24.3)		920 (34.6)	1011 (34.1)	
ARB, *n* (%)							.014			.046
No	5387 (95.7)	1083 (95.8)	1078 (94.7)	1051 (94.8)	1082 (96.0)	1093 (97.3)		2529 (95.1)	2858 (96.3)	
Yes	240 (4.3)	47 (4.2)	60 (5.3)	58 (5.2)	45 (4.0)	30 (2.7)		129 (4.9)	111 (3.7)	
Diuretics, *n* (%)							.013			<.001
No	4809 (85.5)	972 (86.0)	957 (84.1)	967 (87.2)	935 (83.0)	978 (87.1)		2198 (82.7)	2611 (87.9)	
Yes	818 (14.5)	158 (14.0)	181 (15.9)	142 (12.8)	192 (17.0)	145 (12.9)		460 (17.3)	358 (12.1)	
PVD, *n* (%)							.835			<.001
No	4549 (80.8)	919 (81.3)	921 (80.9)	896 (80.8)	898 (79.7)	915 (81.5)		2208 (83.1)	2341 (78.8)	
Yes	1078 (19.2)	211 (18.7)	217 (19.1)	213 (19.2)	229 (20.3)	208 (18.5)		450 (16.9)	628 (21.2)	
Hypertension, *n* (%)							.009			.833
No	3553 (63.1)	684 (60.5)	693 (60.9)	718 (64.7)	708 (62.8)	750 (66.8)		1674 (63.0)	1879 (63.3)	
Yes	2074 (36.9)	446 (39.5)	445 (39.1)	391 (35.3)	419 (37.2)	373 (33.2)		984 (37.0)	1090 (36.7)	
COPD, *n* (%)							<.001			.012
No	3550 (63.1)	677 (59.9)	681 (59.8)	710 (64.0)	717 (63.6)	765 (68.1)		1631 (61.4)	1919 (64.6)	
Yes	2077 (36.9)	453 (40.1)	457 (40.2)	399 (36.0)	410 (36.4)	358 (31.9)		1027 (38.6)	1050 (35.4)	
Diabetes, *n* (%)							.112			<.001
No	3272 (58.1)	665 (58.8)	666 (58.5)	623 (56.2)	687 (61.0)	631 (56.2)		1303 (49.0)	1969 (66.3)	
Yes	2355 (41.9)	465 (41.2)	472 (41.5)	486 (43.8)	440 (39.0)	492 (43.8)		1355 (51.0)	1000 (33.7)	
Renal failure, *n* (%)							.153			.047
No	3425 (60.9)	671 (59.4)	712 (62.6)	697 (62.8)	687 (61.0)	658 (58.6)		1581 (59.5)	1844 (62.1)	
Yes	2202 (39.1)	459 (40.6)	426 (37.4)	412 (37.2)	440 (39.0)	465 (41.4)		1077 (40.5)	1125 (37.9)	
AF, *n* (%)							<.001			.445
No	4139 (73.6)	901 (79.7)	862 (75.7)	770 (69.4)	791 (70.2)	815 (72.6)		1942 (73.1)	2197 (74.0)	
Yes	1488 (26.4)	229 (20.3)	276 (24.3)	339 (30.6)	336 (29.8)	308 (27.4)		716 (26.9)	772 (26.0)	
MI, *n* (%)							.801			.507
No	3598 (63.9)	735 (65.0)	728 (64.0)	693 (62.5)	721 (64.0)	721 (64.2)		1712 (64.4)	1886 (63.5)	
Yes	2029 (36.1)	395 (35.0)	410 (36.0)	416 (37.5)	406 (36.0)	402 (35.8)		946 (35.6)	1083 (36.5)	
Respiratory rate, mean (SD)	19.91 (6.85)	18.08 (5.97)	19.09 (6.10)	19.82 (6.01)	20.91 (6.69)	21.67 (8.56)	<.001	19.76 (6.30)	20.04 (7.31)	.115
Heart rate, mean (SD)	89.33 (20.10)	83.97 (17.48)	87.21 (18.42)	88.23 (18.69)	91.90 (20.90)	95.38 (22.68)	<.001	89.09 (19.93)	89.55 (20.26)	.396
SBP (mmHg), mean (SD)	118.81 (24.52)	120.51 (23.76)	119.29 (24.30)	119.57 (24.77)	118.93 (25.00)	115.75 (24.54)	<.001	118.46 (24.23)	119.12 (24.78)	.315
DBP (mmHg), mean (SD)	65.69 (21.25)	62.53 (15.82)	65.47 (30.19)	66.16 (18.05)	67.77 (19.31)	66.53 (19.45)	<.001	65.78 (24.51)	65.61 (17.84)	.768
SPO_2_, mean (SD)	96.92 (16.97)	97.29 (3.75)	96.97 (3.93)	96.94 (3.64)	97.62 (36.80)	95.80 (6.36)	.119	97.21 (24.13)	96.67 (4.95)	.232
Hemoglobin (g/dL), mean (SD)	10.97 (2.27)	11.05 (2.03)	11.08 (2.21)	11.12 (2.26)	10.89 (2.33)	10.69 (2.45)	<.001	11.15 (2.30)	10.81 (2.23)	<.001
Neutrophil, mean (SD)	78.41 (12.09)	77.02 (11.84)	77.96 (11.05)	77.01 (13.25)	80.07 (11.91)	79.99 (11.94)	<.001	77.98 (11.76)	78.80 (12.36)	.011
Lymphocytes, mean (SD)	12.38 (9.67)	14.51 (9.79)	13.54 (9.55)	13.75 (10.97)	10.62 (8.63)	9.49 (8.21)	<.001	12.62 (9.41)	12.17 (9.90)	.078
Hematocrit (%), mean (SD)	33.54 (6.69)	33.61 (5.89)	33.65 (6.37)	33.86 (6.70)	33.55 (7.10)	33.05 (7.27)	.061	34.17 (6.78)	32.98 (6.55)	<.001
Albumin, mean (SD)	3.23 (0.65)	3.65 (0.51)	3.38 (0.56)	3.26 (0.58)	3.05 (0.60)	2.78 (0.63)	<.001	3.29 (0.64)	3.17 (0.65)	<.001
BUN (mg/dL), mean (SD)	35.18 (24.40)	33.15 (22.27)	32.57 (22.84)	33.00 (23.02)	37.19 (26.03)	39.99 (26.68)	<.001	35.25 (24.11)	35.11 (24.66)	.828
Creatinine (mg/dl), mean (SD)	1.81 (1.75)	1.81 (1.87)	1.67 (1.60)	1.71 (1.62)	1.75 (1.46)	2.13 (2.08)	<.001	1.82 (1.59)	1.80 (1.88)	.645
Creatine kinase, mean (SD)	547.31 (1951.58)	373.74 (871.74)	359.37 (979.35)	537.92 (2123.90)	525.91 (1554.42)	943.16 (3202.80)	<.001	554.67 (1849.83)	540.72 (2038.66)	.789
Potassium (mmol/L), mean (SD)	4.32 (0.79)	4.28 (0.64)	4.25 (0.71)	4.27 (0.72)	4.31 (0.81)	4.48 (1.01)	<.001	4.34 (0.76)	4.30 (0.82)	.117
Sodium (mmol/L), mean (SD)	138.12 (5.45)	138.82 (4.44)	138.10 (5.20)	138.07 (5.05)	137.91 (5.64)	137.71 (6.61)	<.001	138.15 (5.15)	138.09 (5.70)	.675
pH, mean (SD)	7.37 (0.14)	7.39 (0.08)	7.39 (0.08)	7.39 (0.08)	7.37 (0.24)	7.32 (0.12)	<.001	7.37 (0.10)	7.37 (0.17)	.017
Lactate, mean (SD)	2.08 (1.80)	0.92 (0.19)	1.26 (0.23)	1.62 (0.30)	2.12 (0.48)	4.50 (2.76)	<.001	2.03 (1.72)	2.13 (1.87)	.035
Glucose (mg/dL), mean (SD)	150.28 (80.93)	132.27 (57.31)	141.03 (62.57)	147.95 (78.31)	155.40 (88.04)	174.96 (103.34)	<.001	156.74 (82.01)	144.51 (79.52)	<.001
PO_2_, mean (SD)	166.34 (134.50)	220.06 (147.23)	195.93 (140.14)	167.76 (135.44)	127.02 (110.16)	120.33 (106.01)	<.001	163.74 (130.64)	168.66 (137.85)	.171
PCO_2_, mean (SD)	42.99 (13.09)	45.01 (14.52)	43.39 (12.44)	42.44 (11.49)	42.24 (12.49)	41.88 (14.02)	<.001	44.74 (13.25)	41.44 (12.75)	<.001
BE (mEq/L), mean (SD)	−0.58 (7.74)	1.46 (4.49)	0.58 (4.63)	0.24 (4.82)	−0.89 (13.20)	−4.33 (6.33)	<.001	−0.17 (5.41)	−0.95 (9.33)	<.001
GCS, mean (SD)	14.22 (2.37)	14.07 (2.76)	14.26 (2.43)	14.15 (2.55)	14.41 (1.85)	14.21 (2.17)	.014	14.23 (2.38)	14.21 (2.37)	.666
OASIS, mean (SD)	37.02 (9.48)	34.92 (8.75)	35.78 (8.99)	36.00 (9.11)	37.56 (9.56)	40.84 (9.80)	<.001	36.72 (9.46)	37.28 (9.50)	.027
SAPS, mean (SD)	42.75 (14.15)	39.88 (12.75)	40.45 (12.98)	40.82 (12.97)	43.31 (13.85)	49.33 (15.83)	<.001	41.89 (13.92)	43.53 (14.32)	<.001
SOFA, mean (SD)	2.87 (2.60)	2.76 (2.38)	2.57 (2.41)	2.71 (2.48)	2.79 (2.53)	3.49 (3.04)	<.001	2.93 (2.64)	2.80 (2.56)	.065

Abbreviations: ACEI, β‐blocker, angiotensin‐converting‐enzyme inhibitor; AF, atrial fibrillation; ARB, angiotensin receptor blocker; BE, base excess; BMI, body mass index; BUN, blood urea nitrogen; COPD, chronic obstructive pulmonary disease; DBP, diastolic blood pressure; GCS, Glasgow Coma Scale; MI, myocardial infarction; OASIS, Oxford Acute Severity of Illness; PCO_2_, partial pressure of carbon dioxide; PO_2_, partial pressure of oxygen; PVD, peripheral vascular disease; SAPS, Simplified Acute Physiology Score; SBP, systolic blood pressure; SOFA, Sequential Organ Failure Assessment; SPO_2_, saturation of peripheral oxygen.

### 3.2 Association between L/A ratio and 28‐day and 1‐year all‐cause mortality

**Figure 2:**
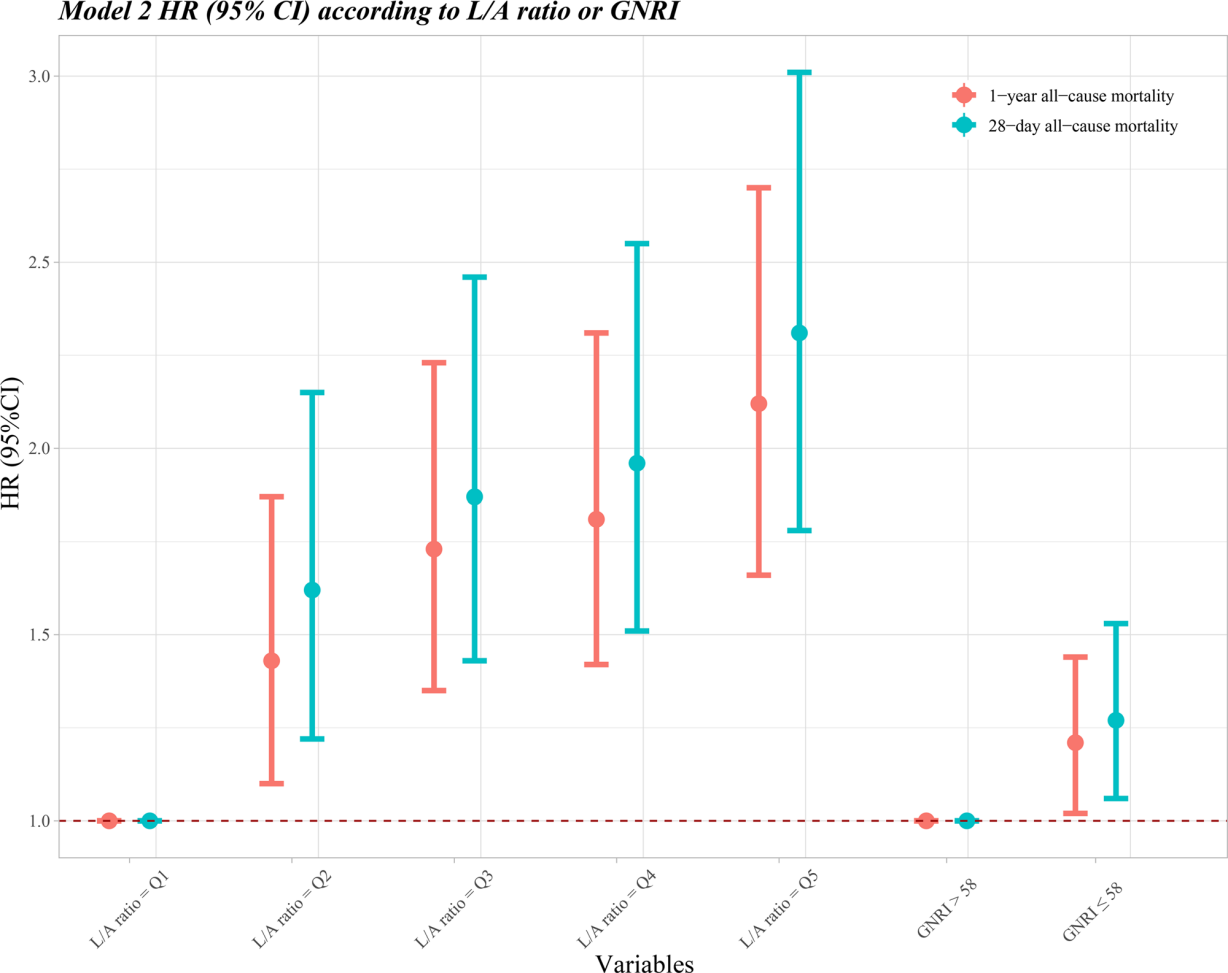
Forest plot for the HR of 28‐day and 1‐year all‐cause mortality according to L/A ratio or GNRI. CI, confidence interval; GNRI, geriatric nutritional risk index; HR, hazard ratio; L/A, lactate/albumin.

KM curve showed a statistical significance between the five groups in 28‐day all‐cause mortality (Supporting Information: Figure 2A) and 1‐year all‐cause mortality (Supporting Information: Figure 2B) (both log‐rank *p* < .001). Results of Cox proportional‐hazards model were displayed in Supporting Information: Table S1. In the unadjusted model, patients in the Q2, Q3, Q4, and Q5 of L/A ratio group had higher risk of 28‐day all‐cause mortality than those in the Q1 of L/A ratio group (all *p* < .001). After adjusting the confounders, similar results were found, with HR of 1.62 (95% CI: 1.22–2.15) for Q2, 1.87 (95% CI: 1.43–2.46) for Q3, 1.96 (95% CI: 1.51–2.55) for Q4, and 2.31 (95% CI: 1.78–3.01) for Q5, respectively. For 1‐year all‐cause mortality, patients in the Q2 (HR: 1.43, 95% CI: 1.10–1.87), Q3 (HR: 1.73, 95% CI: 1.35–2.23), Q4 (HR: 1.81, 95% CI: 1.42–2.31), and Q5 (HR: 2.12, 95% CI: 1.66–2.70) had higher risk than those in the Q1 of L/A ratio group after adjustment of confounding factors. The forest plot for the HR of 28‐day and 1‐year all‐cause mortality according to L/A ratio was shown in Figure 2.

### 3.3 Association between GNRI score and 28‐day and 1‐year all‐cause mortality

Through KM curve, we found the cumulative 28‐day and 1‐year all‐cause mortality between GNRI > 58 group and GNRI ≤ 58 group were significantly different over time (log‐rank *p* < .001 and log‐rank *p* = .001, respectively) (Supporting Information: Figure 3A,B). Supporting Information: Table S1 shows that patients with GNRI ≤ 58 had higher risk of 28‐day all‐cause mortality (HR: 1.24, 95% CI: 1.10–1.41) and 1‐year all‐cause mortality (HR: 1.21, 95% CI: 1.08–‐1.36). After adjusting age, marital status, ethnicity, BMI, ARB, diabetes, renal failure, hemoglobin, hematocrit, glucose, OASIS, SAPS, the results were similar, and HR was 1.27 (95% CI: 1.06–1.53) and 1.21 (95% CI: 1.02–1.44), respectively. The forest plot for the HR of 28‐day and 1‐year all‐cause mortality according to GNRI was shown in Figure 2.

### 3.4 The interaction effect of L/A ratio and GNRI score on 28‐day and 1‐year all‐cause mortality

**Figure 3:**
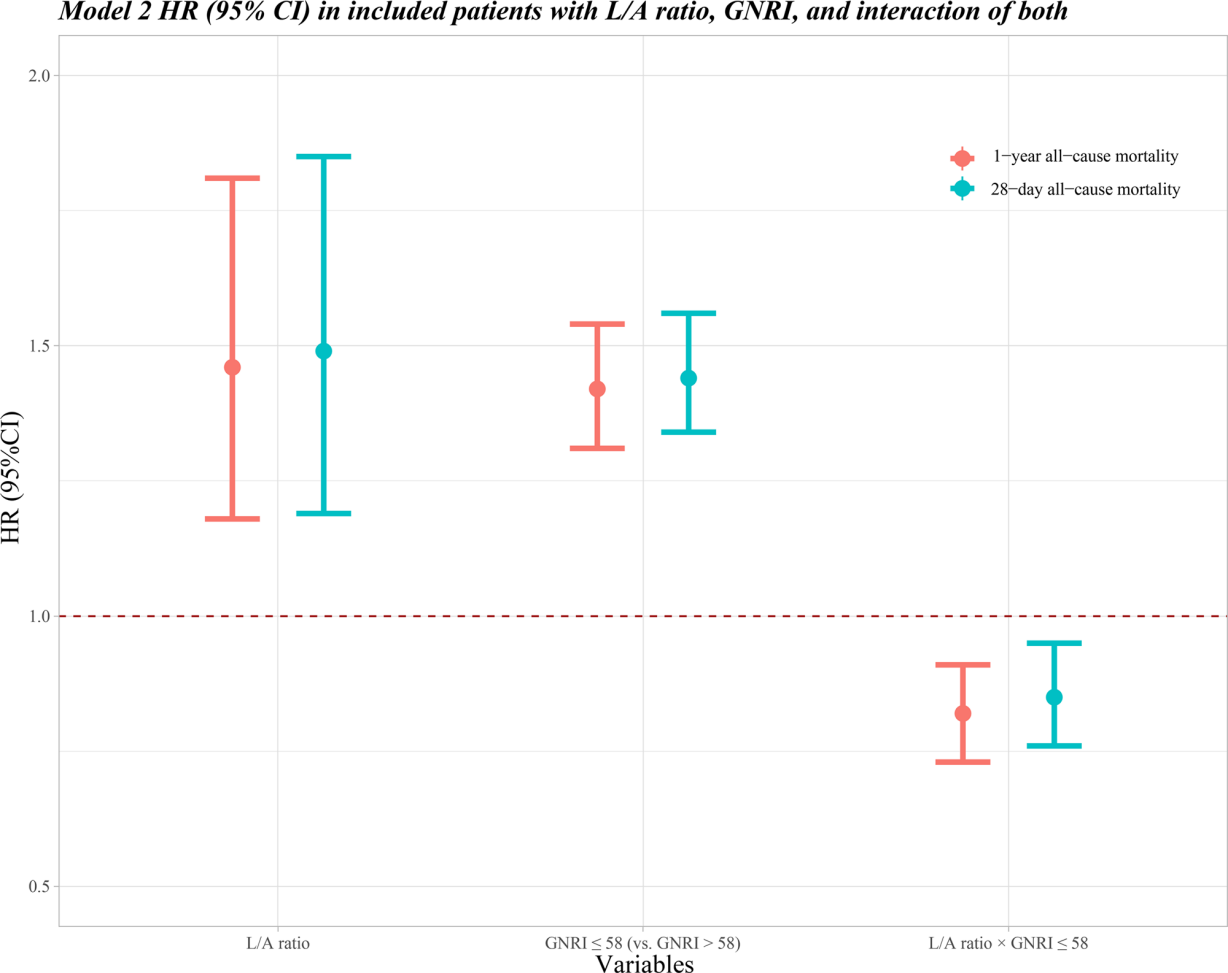
Forest plot for the significance of interaction of L/A ratio and GNRI on the 28‐day and 1‐year all‐cause mortality. CI, confidence interval; GNRI, geriatric nutritional risk index; HR, hazard ratio; L/A, lactate/albumin.

**Figure 4:**
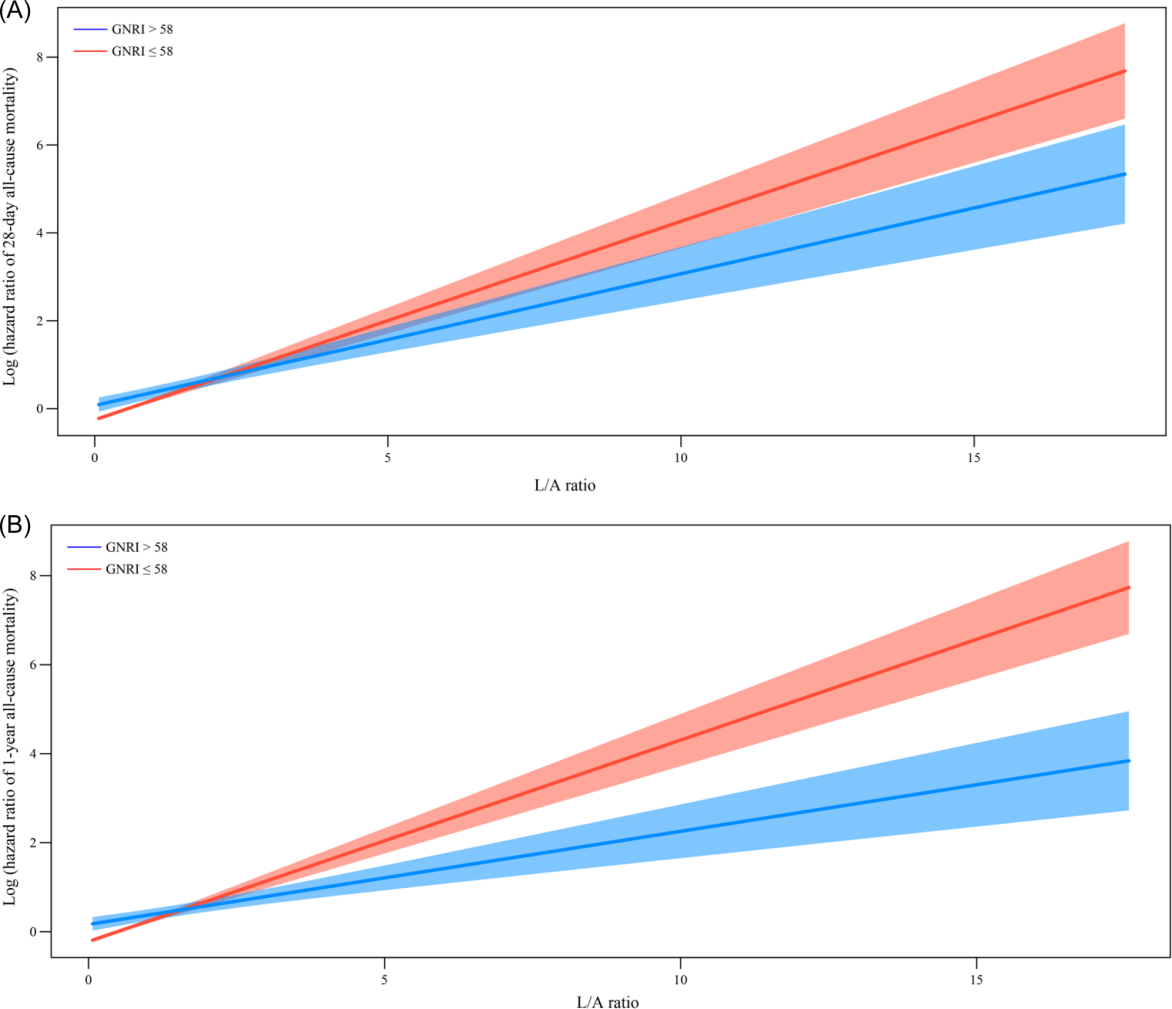
Association between L/A ratio and 28‐day (A) and 1‐year all‐cause mortality (B) in different GNRI groups. GNRI, geriatric nutritional risk index; L/A, lactate/albumin.

After adjusting age, marital status, ethnicity, BMI, ARB, diabetes, renal failure, hemoglobin, hematocrit, glucose, OASIS, and SAPS, the risk of 28‐day all‐cause mortality (HR: 1.49, 95% CI: 1.19–1.85) and 1‐year all‐cause mortality (HR: 1.46, 95% CI: 1.18–1.81) was higher with the increasing L/A ratio. Compared to GNRI > 58, GNRI ≤ 58 was associated with the higher risk of 28‐day and 1‐year all‐cause mortality, with HR of 1.44 (95% CI: 1.34–1.56) and 1.42 (95% CI: 1.31–1.54). Moreover, we found the significant multiplicative interaction effect between L/A ratio and GNRI score for 28‐day and 1‐year all‐cause mortality of critically ill elderly patients with HF (*p* < .05; Supporting Information: Table S2). The forest plot for the significance of the interaction of L/A ratio and GNRI on the 28‐day and 1‐year all‐cause mortality was shown in Figure 3. Figure 4A shows the risk of 28‐day all‐cause mortality of critically ill elderly patients with HF was higher in GNRI ≤ 58 group than the score >58 group as the L/A ratio increased. The result was similar in 1‐year all‐cause mortality; with the increase of L/A ratio, GNRI ≤ 58 displayed the higher risk than the score >58 (Figure 4B).

## 4 DISCUSSION

Critically ill elderly patients with HF have a high mortality, which poses a high burden to the economy and medical resources.^3^, ^4^ Thus, predicting the risk of mortality of these patients can help clinicians make relevant clinical decisions earlier. In this study, we evaluated the association and multiplicative interaction of L/A ratio and GNRI score on the 28‐day and 1‐year all‐cause mortality of critically ill elderly patients with HF. The results showed that higher L/A ratio was associated with higher risk of 28‐day and 1‐year all‐cause mortality. Patients with low GNRI scores had an increased risk of 28‐day and 1‐year all‐cause mortality compared to those with the high score. Also, we found the significant multiplicative interaction effect between L/A ratio and GNRI on the 28‐day and 1‐year all‐cause mortality. With the increase of L/A ratio, the risk of 28‐day and 1‐year all‐cause mortality were higher in low GNRI score group than the high score group.

L/A ratio has been reported as an effective prognostic marker than lactate or albumin alone for critically ill patients.^9^ In our study, higher L/A ratio was associated with the higher risk of short‐term and long‐term all‐cause mortality. The positive association between L/A ratio and the risk of all‐cause mortality in critically ill patients with HF may be explained as follows. The level of lactate may be increased by insufficient tissue perfusion and anaerobic metabolism in critically ill HF patients resulting from poor cardiac function or long‐term and large‐scale use of diuretics.^19^, ^20^ Evidence has showed the positive association between the increased lactate level and the elevated risk of 1‐year mortality in patients with HF.^5^ In HF patients, lactate level is a marker of hypoperfusion and hypoxia, and elevated level is associated with hemodynamic instability.^5^ Patients with severe HF had chronic consumption or liver congestion, which may result in hypoalbuminemia.^21^ Previous studies have showed that approximately 14% of HF patients had hypoalbuminemia, and hypoalbuminemia caused an increased risk of death.^21^, ^22^ Therefore, a high L/A ratio reflects an increase of lactate level and a decrease of albumin level, which elevates the risk of all‐cause mortality in severe HF patients.

The nutritional guidelines have emphasized the nutrition screening for critical ill patients.^23^, ^24^ In elderly HF patients, cardiac dysfunction and venous congestion predispose to bowel edema and malabsorption, thereby resulting in malnutrition.^25^ Malnutrition is highly prevalent in elderly HF patients and associated with the higher death rate.^26^, ^27^ GNRI is a valuable and objective indicator for risk of malnutritional status of elderly patients, and lower GNRI means the worse nutritional status.^25^ In our study, we found that the low GNRI score was correlated with higher all‐cause mortality, which was consistent with the results of previously studies.^16^, ^25^, ^28^ The following mechanisms may explain the association between GNRI and mortality. GNRI is calculated by albumin, actual weight, and ideal weight.^18^ Hypoalbuminemia is recognized as a factor contributing to developing HF.^29^ Both hypoalbuminemia^30^ and weight loss^31^ displayed an effective and consistent outcome‐predictability of death. Therefore, GNRI that combines albumin, actual weight, and ideal weight can show a synergistical effect on mortality. In our study, a significant multiplicative interaction effect between L/A ratio and GNRI score was observed, implying that GNRI score affected the association between L/A ratio and mortality. Further, we found the risk of mortality caused by the increased L/A ratio was higher in the low GNRI score group than the high score group. This finding suggested that more attention on the nutritional status is needed in elderly patients with high L/A levels.

Our study explores the association and multiplicative interaction of L/A ratio and GNRI on the long‐term and short‐term all‐cause mortality of critically ill elderly patients with HF. Some limitations are existed in our study. First, our data were extracted from the MIMIC‐III database and data missing is inevitable, but the missing data have been imputed using multiple imputations. Second, the lactate level is collected at baseline; therefore, we cannot assess the impact of dynamic change of L/A ratio on the risk of mortality.

## 5 CONCLUSION

In conclusion, our study found the association and multiplicative interaction of L/A ratio and GNRI score on the all‐cause mortality of critically ill elderly HF patients, and increased L/A ratio was associated with the higher risk of all‐cause mortality in the low GNRI score group. Our findings emphasized the importance of nutrition‐oriented management and intervention in reducing the risk of mortality among elderly HF patients with high L/A ratio in the ICU.

## CONFLICT OF INTEREST STATEMENT

The authors declare no conflict of interest.

## Supporting information

Figurementary figure 1.

Supporting information.

Figurementary figure 2.

Supporting information.

Figurementary figure 3.

Supporting information.

Supporting information.

## Data Availability

The datasets used and/or analyzed during the current study are available from the corresponding author on reasonable request.

## References

[1] Chang PP , Wruck LM , Shahar E , et al. Trends in hospitalizations and survival of acute decompensated heart failure in four US communities (2005‐2014): ARIC study community surveillance. Circulation. 2018;138(1):12‐24. 29519849 10.1161/CIRCULATIONAHA.117.027551 PMC6030442

[2] Tsao CW , Aday AW , Almarzooq ZI , et al. Heart disease and stroke statistics‐2022 update: a report from the American Heart Association. Circulation. 2022;145(8):e153‐e639. 35078371 10.1161/CIR.0000000000001052

[3] Jentzer JC , Reddy YN , Rosenbaum AN , et al. Outcomes and predictors of mortality among cardiac intensive care unit patients with heart failure. J Card Failure. 2022;28( 7):1088‐1099. 10.1016/j.cardfail.2022.02.015 35381356

[4] Dharmarajan K , Rich MW . Epidemiology, pathophysiology, and prognosis of heart failure in older adults. Heart Fail Clin. 2017;13(3):417‐426. 28602363 10.1016/j.hfc.2017.02.001

[5] Zymliński R , Biegus J , Sokolski M , et al. Increased blood lactate is prevalent and identifies poor prognosis in patients with acute heart failure without overt peripheral hypoperfusion. Eur J Heart Fail. 2018;20(6):1011‐1018. 29431284 10.1002/ejhf.1156

[6] Haas SA , Lange T , Saugel B , et al. Severe hyperlactatemia, lactate clearance and mortality in unselected critically ill patients. Intensive Care Med. 2016;42(2):202‐210. 26556617 10.1007/s00134-015-4127-0

[7] Ancion A , Allepaerts S , Oury C , Gori AS , Piérard LA , Lancellotti P . Serum albumin level and hospital mortality in acute non‐ischemic heart failure. ESC Heart Fail. 2017;4(2):138‐145. 28451450 10.1002/ehf2.12128 PMC5396050

[8] Gharipour A , Razavi R , Gharipour M , Mukasa D . Lactate/albumin ratio: an early prognostic marker in critically ill patients. Am J Emerg Med. 2020;38(10):2088‐2095. 33152585 10.1016/j.ajem.2020.06.067

[9] Cakir E , Turan IO . Lactate/albumin ratio is more effective than lactate or albumin alone in predicting clinical outcomes in intensive care patients with sepsis. Scand J Clin Lab Invest. 2021;81(3):225‐229. 33745405 10.1080/00365513.2021.1901306

[10] Guo W , Zhao L , Zhao H , et al. The value of lactate/albumin ratio for predicting the clinical outcomes of critically ill patients with heart failure. Ann Transl Med. 2021;9(2):118. 33569420 10.21037/atm-20-4519 PMC7867948

[11] Shpata V , Ohri I , Nurka T , Prendushi X . The prevalence and consequences of malnutrition risk in elderly Albanian intensive care unit patients. Clin Interv Aging. 2015;10:481‐486. 25733824 10.2147/CIA.S77042 PMC4337415

[12] Honda Y , Nagai T , Iwakami N , et al. Usefulness of geriatric nutritional risk index for assessing nutritional status and its prognostic impact in patients aged ≥65 years with acute heart failure. Am J Cardiol. 2016;118(4):550‐555. 27324158 10.1016/j.amjcard.2016.05.045

[13] Sargento L , Vicente Simões A , Rodrigues J , Longo S , Lousada N , Palma dos Reis R . Geriatric nutritional risk index as a nutritional and survival risk assessment tool in stable outpatients with systolic heart failure. Nutr Metab Cardiovasc Dis. 2017;27(5):430‐437. 28438373 10.1016/j.numecd.2017.02.003

[14] Sze S , Pellicori P , Kazmi S , et al. Prevalence and prognostic significance of malnutrition using 3 scoring systems among outpatients with heart failure. JACC Heart Fail. 2018;6(6):476‐486. 29753673 10.1016/j.jchf.2018.02.018

[15] Alataş ÖD , Biteker M , Yildirim B , Acar E , Gökçek K . Comparison of objective nutritional indexes for the prediction of in‐hospital mortality among elderly patients with acute heart failure. Eur J Emerg Med. 2020;27(5):362‐367. 32217850 10.1097/MEJ.0000000000000690

[16] Nishi I , Seo Y , Hamada‐Harimura Y , et al. Geriatric nutritional risk index predicts all‐cause deaths in heart failure with preserved ejection fraction. ESC Heart Fail. 2019;6(2):396‐405. 30706996 10.1002/ehf2.12405 PMC6437432

[17] Johnson AEW , Pollard TJ , Shen L , et al. MIMIC‐III, a freely accessible critical care database. Sci Data. 2016;3:160035. 27219127 10.1038/sdata.2016.35 PMC4878278

[18] Bouillanne O , Morineau G , Dupont C , et al. Geriatric nutritional risk index: a new index for evaluating at‐risk elderly medical patients. Am J Clin Nutr. 2005;82(4):777‐783. 16210706 10.1093/ajcn/82.4.777

[19] Andersen LW , Mackenhauer J , Roberts JC , Berg KM , Cocchi MN , Donnino MW . Etiology and therapeutic approach to elevated lactate levels. Mayo Clin Proc. 2013;88(10):1127‐1140. 24079682 10.1016/j.mayocp.2013.06.012 PMC3975915

[20] Smith ZR , Horng M , Rech MA . Medication‐induced hyperlactatemia and lactic acidosis: a systematic review of the literature. Pharmacotherapy. 2019;39(9):946‐963. 31361914 10.1002/phar.2316

[21] Liu M , Chan CP , Yan BP , et al. Albumin levels predict survival in patients with heart failure and preserved ejection fraction. Eur J Heart Fail. 2012;14(1):39‐44. 22158777 10.1093/eurjhf/hfr154

[22] Kempny A , Diller GP , Alonso‐Gonzalez R , et al. Hypoalbuminaemia predicts outcome in adult patients with congenital heart disease. Heart. 2015;101(9):699‐705. 25736048 10.1136/heartjnl-2014-306970 PMC4413739

[23] McClave SA , Taylor BE , Martindale RG , et al. Guidelines for the provision and assessment of nutrition support therapy in the adult critically ill patient: Society of Critical Care Medicine (SCCM) and American Society for Parenteral and Enteral Nutrition (A.S.P.E.N.). JPEN J Parenter Enteral Nutr. 2016;40(2):159‐211. 26773077 10.1177/0148607115621863

[24] Mesejo A , Vaquerizo Alonso C , Acosta Escribano J . [Guidelines for specialized nutritional and metabolic support in the critically‐ill patient. Update. Consensus of the Spanish Society of Intensive Care Medicine and Coronary Units‐Spanish Society of Parenteral and Enteral Nutrition (SEMICYUC‐SENPE): macro‐and micronutrient requirements]. Med Intensiva. 2011;35(suppl 1):1‐6. 10.1016/S0210-5691(11)70015-8 22309758

[25] Sze S , Pellicori P , Zhang J , Clark AL . Malnutrition, congestion and mortality in ambulatory patients with heart failure. Heart. 2019;105(4):297‐306. 30121635 10.1136/heartjnl-2018-313312

[26] Kinugawa S , Fukushima A . Malnutrition in heart failure. JACC Heart Fail. 2018;6(6):487‐488. 29753674 10.1016/j.jchf.2018.03.014

[27] Hirose S , Matsue Y , Kamiya K , et al. Prevalence and prognostic implications of malnutrition as defined by GLIM criteria in elderly patients with heart failure. Clin Nutr. 2021;40(6):4334‐4340. 33551220 10.1016/j.clnu.2021.01.014

[28] Minamisawa M , Seidelmann SB , Claggett B , et al. Impact of malnutrition using geriatric nutritional risk index in heart failure with preserved ejection fraction. JACC Heart Fail. 2019;7(8):664‐675. 31302049 10.1016/j.jchf.2019.04.020 PMC13537618

[29] Gopal DM , Kalogeropoulos AP , Georgiopoulou VV , et al. Serum albumin concentration and heart failure risk. Am Heart J. 2010;160(2):279‐285. 20691833 10.1016/j.ahj.2010.05.022 PMC2919495

[30] Peng W , Zhang C , Wang Z , Yang W . Prediction of all‐cause mortality with hypoalbuminemia in patients with heart failure: a meta‐analysis. Biomarkers. 2019;24(7):631‐637. 31379211 10.1080/1354750X.2019.1652686

[31] Fan Y , Gu X , Zou C . Prediction of all‐cause and cardiovascular mortality with weight loss in patients with chronic heart failure: a meta‐analysis. Eur J Prev Cardiol. 2020;27(19):2155‐2158. 31412711 10.1177/2047487319871122

