# Supplementary figures and images for: Interaction of lactate/albumin and geriatric nutritional risk index on the all‐cause mortality of elderly patients with critically ill heart failure: A cohort study
Source: Clin Cardiol. 2023 May 24;46(7):745–56. doi: 10.1002/clc.24029 (PMC10352977; doi:10.1002/clc.24029)

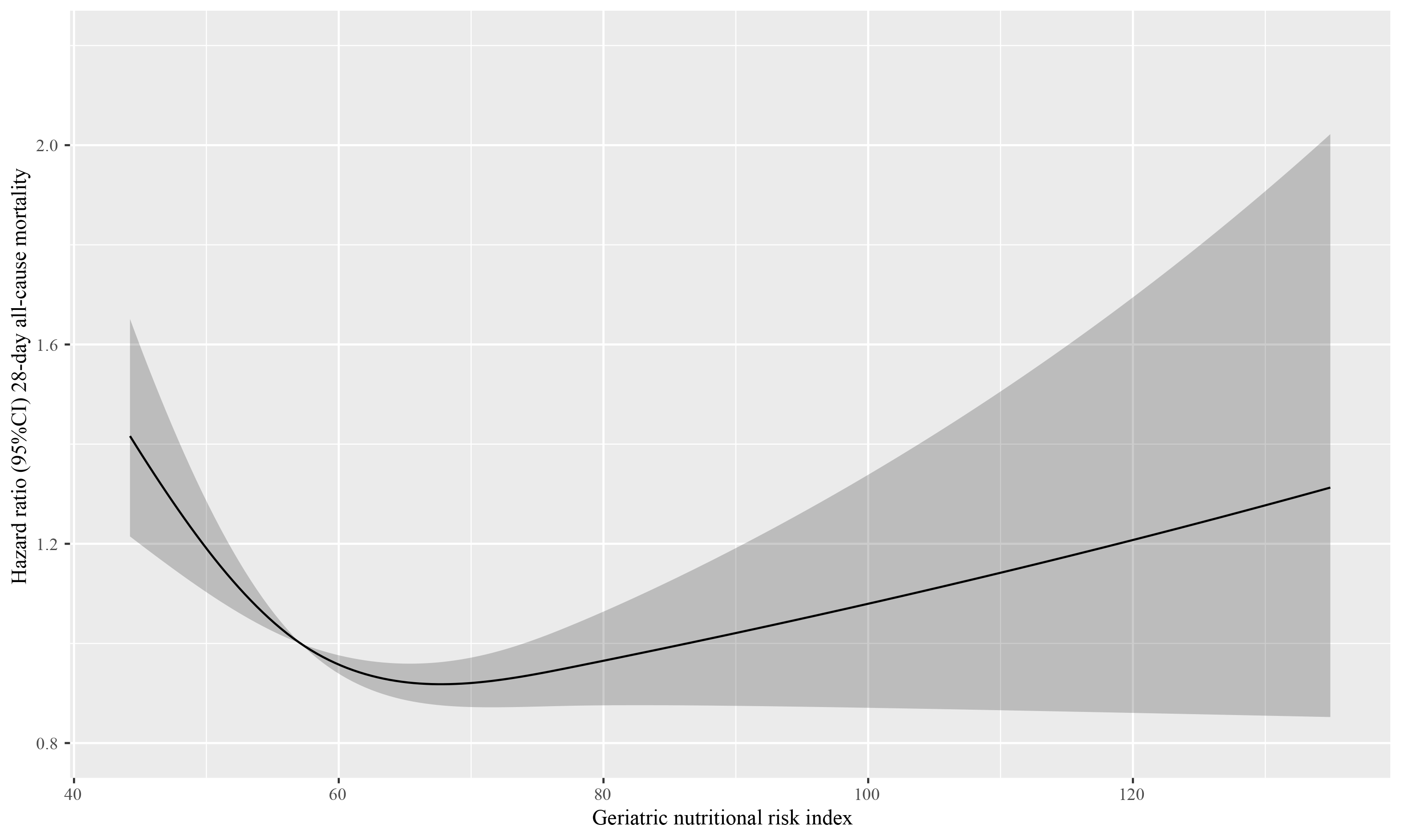

Supplement: Supplementary file 1 — Figurementary figure 1. [file CLC-46-745-s001.tif]

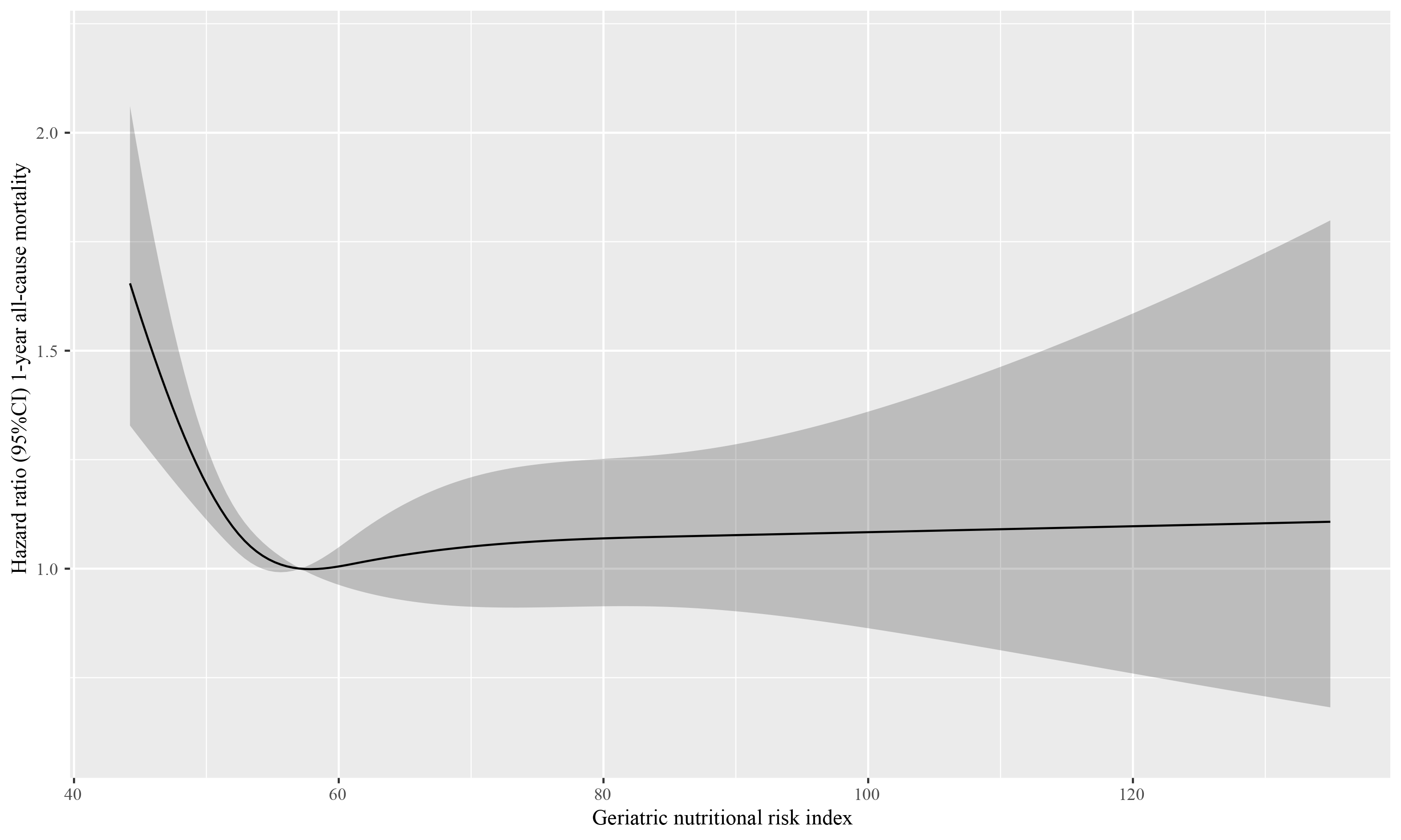

Supplement: Supplementary file 2 — Supporting information. [file CLC-46-745-s003.tif]

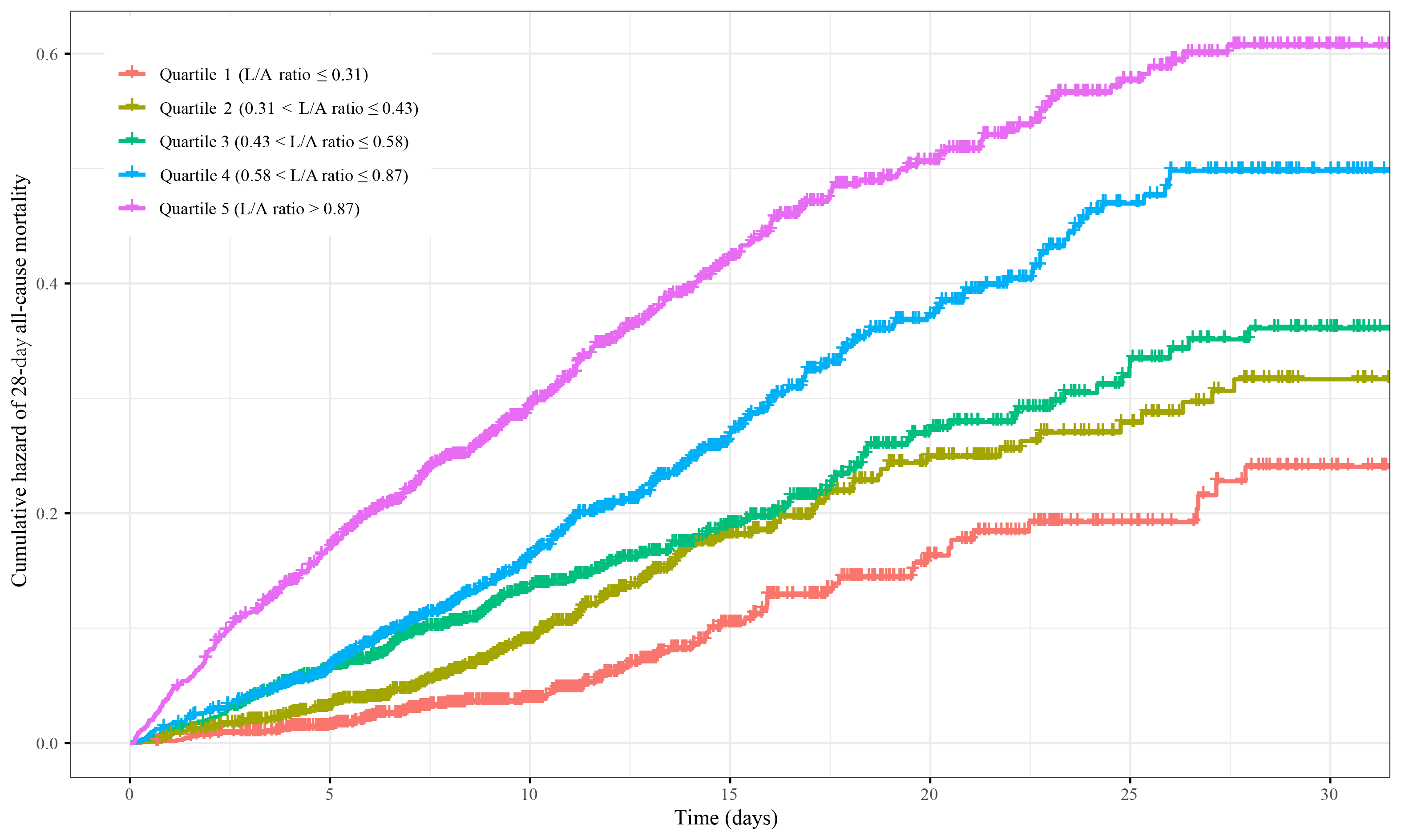

Supplement: Supplementary file 3 — Figurementary figure 2. [file CLC-46-745-s005.tif]

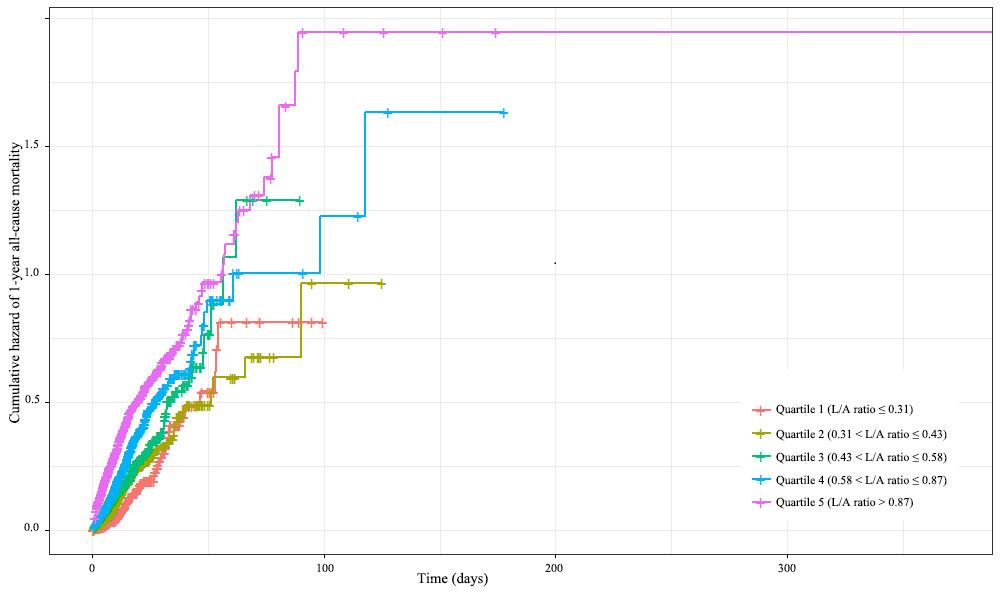

Supplement: Supplementary file 4 — Supporting information. [file CLC-46-745-s004.tif]

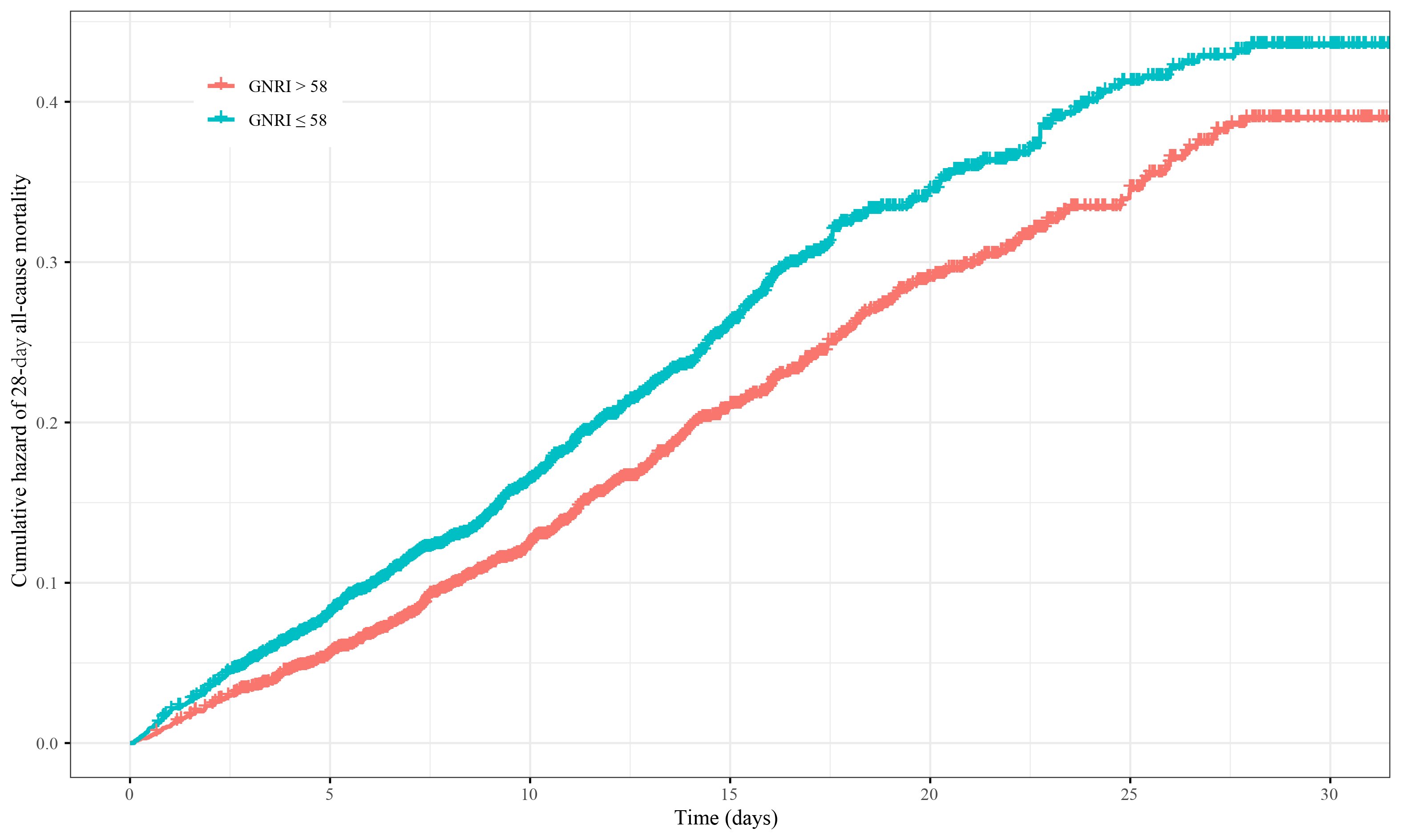

Supplement: Supplementary file 5 — Figurementary figure 3. [file CLC-46-745-s002.tif]

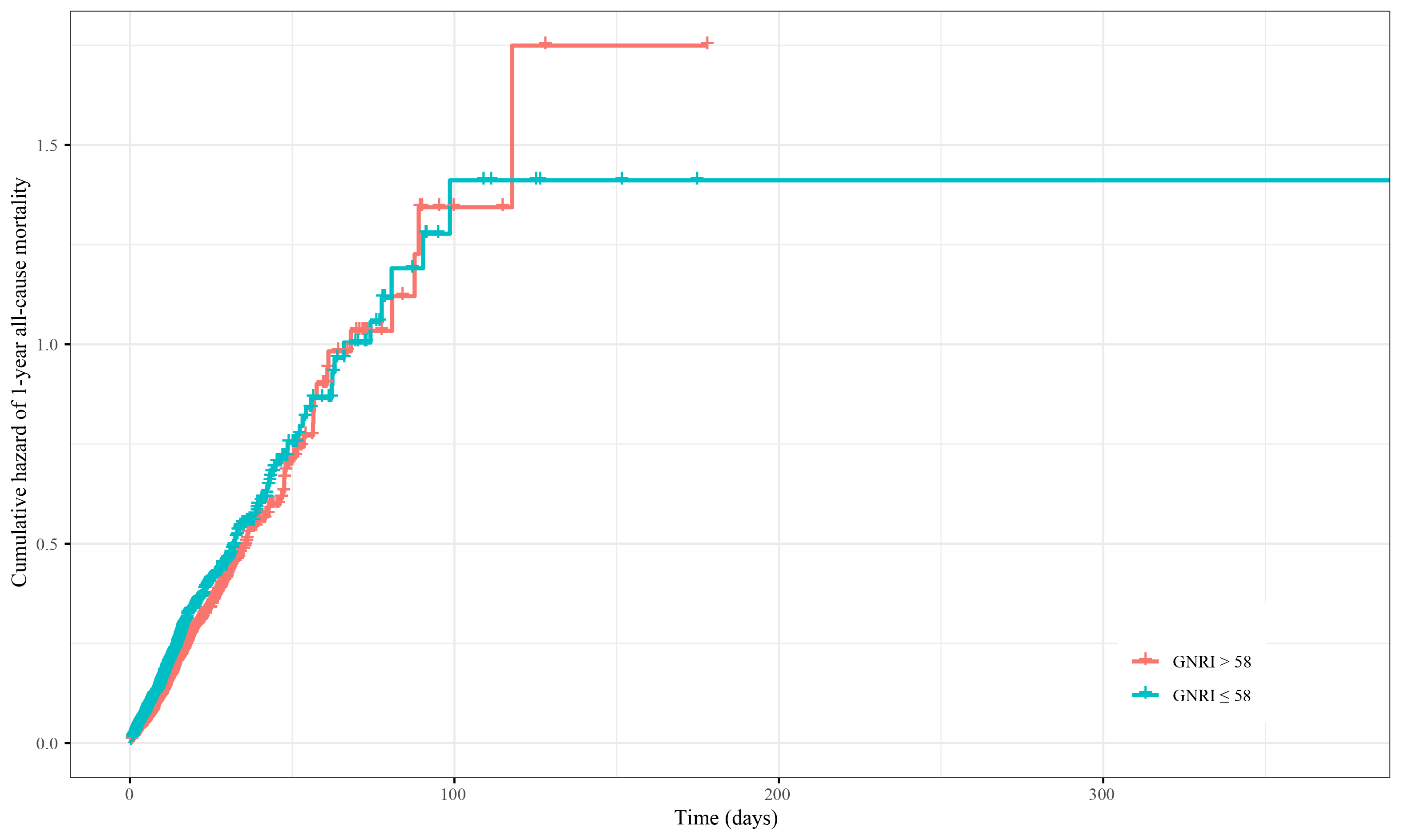

Supplement: Supplementary file 6 — Supporting information. [file CLC-46-745-s007.tif]
