# Supplementary material for: Interaction of lactate/albumin and geriatric nutritional risk index on the all‐cause mortality of elderly patients with critically ill heart failure: A cohort study
Source: Clin Cardiol. 2023 May 24;46(7):745–56. doi: 10.1002/clc.24029 (PMC10352977; doi:10.1002/clc.24029)
Supplement: Supplementary file 7 — Supporting information. [file CLC-46-745-s006.docx]

**Supplementary table S1 HR (95% CI) of 28-day all-cause mortality and 1-year all-cause mortality according to L/A ratio or GNRI**

|  | **28-day all-cause mortality** | | **1-year all-cause mortality** | |
| --- | --- | --- | --- | --- |
|  | **Model 1** | **Model 2** | **Model 1** | **Model 2** |
|  | **HR (95%CI)** | **HR (95%CI)** | **HR (95%CI)** | **HR (95%CI)** |
| L/A ratio quintile |  |  |  |  |
| Q1 | Reference | Reference | Reference | Reference |
| Q2 | 1.67 (1.26-2.22) *** | 1.62 (1.22-2.15) *** | 1.49 (1.15-1.94) ** | 1.43 (1.10-1.87) ** |
| Q3 | 2.12 (1.62-2.78) *** | 1.87 (1.43-2.46) *** | 1.97 (1.54-2.53) *** | 1.73 (1.35-2.23) *** |
| Q4 | 2.84 (2.19-3.67) *** | 1.96 (1.51-2.55) *** | 2.50 (1.97-3.17) *** | 1.81 (1.42-2.31) *** |
| Q5 | 4.29 (3.35-5.49) *** | 2.31 (1.78-3.01) *** | 3.69 (2.94-4.64) *** | 2.12 (1.66-2.70) *** |
| GNRI score |  |  |  |  |
| GNRI > 58 | Reference | Reference | Reference | Reference |
| GNRI ≤ 58 | 1.24 (1.10-1.41) *** | 1.27 (1.06-1.53) ** | 1.21 (1.08-1.36) ** | 1.21 (1.02-1.44) * |

L/A, lactate/albumin; GNRI, geriatric nutritional risk index; HR, hazard ratio; CI, confidence interval. * *P* < 0.05, ** *P* < 0.01, *** *P* < 0.001.

Models for L/A ratio quintile: Model 1: univariable analysis; Model 2: multivariable analysis, adjusted for marital status, body mass index (BMI), β-blocker, angiotensin-converting-enzyme inhibitor (ACEI), angiotensin receptor blocker (ARB), hypertension, lymphocytes, blood urea nitrogen (BUN), creatinine, creatine kinase, sodium, partial pressure of oxygen (PO_2_), base excess (BE), Glasgow Coma Scale (GCS), Oxford Acute Severity of Illness (OASIS), Simplified Acute Physiology Score (SAPS), and Sequential Organ Failure Assessment (SOFA).

Models for GNRI score: Model 1: univariable analysis; Model 2: multivariable analysis, adjusted for age, marital status, ethnicity, body mass index (BMI), angiotensin receptor blocker (ARB), diabetes, renal failure, hemoglobin, hematocrit, glucose, Oxford Acute Severity of Illness (OASIS), and Simplified Acute Physiology Score (SAPS).

**Supplementary table S2 HR (95% CI) of 28-day all-cause mortality and 1-year all-cause mortality in included patients with L/A ratio, GNRI, and interaction of both**

| **Variables** | **28-day all-cause mortality** | | | | **1-year all-cause mortality** | | | |
| --- | --- | --- | --- | --- | --- | --- | --- | --- |
|  | **Model 1** | | **Model 2** | | **Model 1** | | **Model 2** | |
|  | **HR (95%CI)** | ***P*** | **HR (95%CI)** | ***P*** | **HR (95%CI)** | ***P*** | **HR (95%CI)** | ***P*** |
| L/A ratio | 1.57 (1.48-1.67) | < 0.001 | 1.49 (1.19-1.85) | < 0.001 | 1.57 (1.48-1.67) | < 0.001 | 1.46 (1.18-1.81) | < 0.001 |
| GNRI ≤ 58 (vs. GNRI > 58) | 1.39 (1.19-1.62) | < 0.001 | 1.44 (1.34-1.56) | < 0.001 | 1.47 (1.27-1.71) | < 0.001 | 1.42 (1.31-1.54) | < 0.001 |
| L/A ratio × GNRI ≤ 58 | 0.86 (0.78-0.94) | 0.001 | 0.85 (0.76-0.95) | 0.003 | 0.78 (0.72-0.86) | < 0.001 | 0.82 (0.73-0.91) | < 0.001 |

L/A, lactate/albumin; GNRI, geriatric nutritional risk index; HR, hazard ratio; CI, confidence interval.

Model 1: univariable analysis;

Model 2: multivariable analysis, adjusted for age, marital status, ethnicity, body mass index (BMI), angiotensin receptor blocker (ARB), diabetes, renal failure, hemoglobin, hematocrit, glucose, Oxford Acute Severity of Illness (OASIS), Simplified Acute Physiology Score (SAPS).
